# Terbinafine Resistance in *Trichophyton* Strains Isolated from Humans and Animals: A Retrospective Cohort Study in Italy, 2016 to May 2024

**DOI:** 10.3390/jcm13185493

**Published:** 2024-09-17

**Authors:** Silvia Crotti, Deborah Cruciani, Michela Sabbatucci, Sara Spina, Vincenzo Piscioneri, Martina Torricelli, Roberta Calcaterra, Claudio Farina, Luigi Pisano, Manuela Papini

**Affiliations:** 1 Istituto Zooprofilattico Sperimentale dell’Umbria e delle Marche “Togo Rosati” (IZSUM), 06126 Perugia, Italy; s.crotti@izsum.it (S.C.); s.spina@izsum.it (S.S.); v.piscioneri@izsum.it (V.P.); m.torricelli@izsum.it (M.T.); 2 Department Infectious Diseases, Istituto Superiore di Sanità, 00161 Rome, Italy; michela.sabbatucci@iss.it; 3 National Institute for Health, Migration and Poverty, 00153 Rome, Italy; roberta.calcaterra@inmp.it; 4 Clinic Microbiology and Virology Laboratory, ASST Papa Giovanni XXIII, 24127 Bergamo, Italy; cfarina@asst-pg23.it; 5 Section of Dermatology, Health Sciences Department, University of Florence, 50100 Florence, Italy; luigi.pisano88@yahoo.it; 6 Clinica Dermatologica di Terni, Dipartimento di Medicina e Chirurgia, Università degli Studi di Perugia, 06123 Perugia, Italy; manuelapapini@tiscali.it

**Keywords:** allylamines, antifungal resistance, antifungal susceptibility, dermatophytes, squalene epoxidase, SQLE, terbinafine, *tinea*

## Abstract

**Background:** In recent decades, globalization and international migration have increased the spread of infectious agents, including dermatophytes. Although considered minor infections, dermatophytoses are highly contagious, and they significantly reduce the quality of life, inducing itching, burning, sleep disturbances, and even depressive states. Moreover, the increasing resistance to antifungals threats the public health and burdens the costs for the healthcare system. **Methods:** DermaGenius^®^ Resistance Multiplex real-time PCR assay allowed to analyze the terbinafine susceptibility/resistance of 172 *Trichophyton* strains, which were isolated from human and animal samples collected from 2016 to May 2024 and previously identified by Sanger sequencing. **Results:** All the 11 animal strains belonged to the *T. interdigitale*/*T. mentagrophytes* complex and tested terbinafine sensitive. Out of 161 human strains, 9 (5.6%) showed terbinafine resistance and 7 (4.3%) were identified as *T. indotineae*. **Conclusions:** This study provides preliminary data about behavior toward antifungals in animals and finalizes the scientific information currently available about human strains, highlighting the importance of the One Health concept. Moreover, it supports the relevant role of *T. indotineae* as an emerging dermatophyte with high proportion of terbinafine resistance.

## 1. Introduction

In the last few decades, globalization and international migration have increased microbial spread and infectious disease transmission, including fungal pathogens and related diseases [1,2,3,4]. Particularly, antifungal-resistant infections represent an increasing health threat worldwide [5,6,7,8]. The extensive use of broad-spectrum antibiotics or prolonged antibiotic therapy, use of invasive devices, prolonged hospital stays, immunodepression, surgery or hemodialysis can favor the emergence of fungal infections that causes substantial clinical impact and cost to the healthcare system.

Dermatophytoses are the most common fungal infections in the world, affecting approximately a quarter of the global population [9,10]. Even if considered mild infections, their high contagiousness may reduce significantly the quality of life of patients by causing itching, burning sensations, depression, stigma, and sleep disturbances [11]. Clinically, dermatophytosis is a superficial fungal infection of the skin exclusively affecting structures rich in keratin material (stratum *corneum*, hair, nails) causing ringworm lesions and alopecic areas, which were traditionally named according to the anatomic locations involved by appending the Latin term of the body site after the word *tinea* (i.e., *tinea capitis*) [12]. The primary pathogens that predominantly infect humans belong to the four genera of *Trichophyton*, *Microsporum*, *Epidermophyton*, and *Nannizzia* [13].

Recently, anthropophilic dermatophytes such as *Epidermophyton* (*E.*) *floccosum*, *Microsporum* (*M.*) *audouinii*, and *Trichophyton* (*T.*) *schoenleinii* saw a decline in prevalence in European countries, being replaced by various *Trichophyton* species [14], most commonly *T. rubrum*, *T. mentagrophytes*, and *T. interdigitale* [15]. In particular, *T. rubrum* is an anthropophilic species responsible for a wide range of fungal infections, including *tinea pedis*, *tinea unguium*, *tinea cruris*, *tinea corporis*, and *tinea manuum* [9]: predominantly, it affects individuals between the age of 20 and 60 with a tendency to infect older patients in recent years [16]. *Trichophyton rubrum* is predominant in Europe, South America, Asia, and Africa with an incidence significantly increased since the 20th century [13].

*Trichophyton mentagrophytes* is considered a zoophilic species and usually manifests as an inflammatory *tinea* of glabrous skin (*tinea corporis*). *Trichophyton interdigitale* is an anthropophilic dermatophyte mainly associated with onychomycosis and *tinea pedis* in humans, while it is absent in animals. The taxonomic classification of *T. mentagrophytes* and *T. interdigitale* has been the subject of much controversy. Švarcová et al. (2023) [17] investigated correlations between molecular identification, clinical manifestation, and morphology for *T. interdigitale* and *T. mentagrophytes*. To avoid confusion and to simplify identification in practice, they recommend using the name *T. mentagrophytes* for the entire complex [17]. In addition, an emerging species named *Trichophyton indotineae* has been recently proposed for *T. mentagrophytes* genotype VIII, which is a predominant cause of the Indian epidemic of superficial mycoses and which frequently shows terbinafine resistance [18]. *Trichophyton indotineae* can be identified by molecular investigations considering that it differs from *T. mentagrophytes* and *T. interdigitale* by only a few single nucleotide polymorphisms (SNPs) in the internal transcribed spacer (ITS) region [19]. *Trichophyton indotineae* emerged in South Asia and has spread rapidly over the past decade to the United States (US) [10,20], South America [21], Australia, China, Canada [22,23], Iran [24], Japan [18], Kuwait [25], Turkey [26], Vietnam [27], and Europe [28,29,30]. Its emergence may have been driven by the improper and excessive use of topical antifungals and corticosteroids. Other potential factors favoring *T. indotineae* infection in developing countries could be a lack of hygiene, overcrowding, working in a hot and humid environment, and wearing tight-fitting synthetic clothing [31]. Moreover, its anthropophilic pattern could promote an inter-human transmission so much that familial cases were detected for about half of the patients, and the sharing of fomites was particularly incriminated [32]. In addition, the intensive migrations and the recovery of tourism since the COVID-19 pandemic could have spread some pathogens, including *T. indotineae*. Infections caused by *T. indotineae* could be severe, causing inflammatory and pruritic forms of difficult-to-treat *tinea cruris*, *tinea corporis*, and *tinea faciei*, which are often resistant to antifungal treatment [18,33,34,35].

Terbinafine or itraconazole [36] therapy can be effective in eradicating infections due to *Trichophyton* spp. However, point mutations in the squalene epoxidase (SQLE) gene inducing resistant forms could frustrate the awaited results. Moreover, even if different methods can be used, antifungal susceptibility testing (AFST) for dermatophytes is not performed routinely, and no breakpoints classifying susceptible or resistant isolates are available yet [34].

Currently, dermatophytosis notifications must be communicated to the national surveillance system of infectious diseases instituted by the Italian Ministerial Decree (MD) 3 March 2017 and regulated by the MD 7 March 2022 “Review of the reporting system for infectious diseases (PREMAL)” [37] that abrogated the previous MD in force since 15 December 1990.

Between 2009 and 2013, there were 1849 cases of dermatophytoses in Italy, including five deaths, which were associated with over 21.000 hospital days [38]. Moreover, in the last few years, dermatophyte cases increased in Italy; i.e., in the Lazio region, there were 3 cases reported in 2020, 14 in 2021, and 84 in 2022 [39]. In the Sicily region, a total of 75 cases of dermatophytoses were notified in the period 2001–2020 [40]. In June 2023, the first *T. indotineae* terbinafine-sensitive case was notified, which was isolated in central Italy from a 42-year-old woman of Indian origin [41].

An epidemiological survey has been conducted on *Trichophyton* spp. strains collected from humans and animals isolated in central Italy between 2016 and 2024 except for one human case collected in northern Italy. The aims of this study were (i) to identify the correct dermatophyte species and (ii) to establish terbinafine resistance or susceptibility of the strains.

## 2. Materials and Methods

This study involved a total of 172 *Trichophyton* strains collected, analyzed, and stored at −80 °C from January 2016 until May 2024: 161 were isolated from human samples and 11 from animals including 6 Eurasian red squirrels (*Sciurus vulgaris*, Linnaeus, 1758), 3 dogs (*Canis lupus familiaris*, Linnaeus, 1758), 1 Eastern gray squirrel (*Sciurus carolinensis*, Gray, 1834), and 1 cat (*Felis catus*, Linnaeus, 1758). Originally, a dermatologist collected skin scraping and nail clipping samples from all subjects showing clinical manifestation compatible with skin or nail fungal infection. There were 77 human onychomycosis cases (47.8%); the other patients were affected by *tinea corporis* (*n* = 38, 23.6%), *tinea pedis* (*n* = 34, 21.1%), *tinea capitis* were (*n* = 4, 2.5%), *tinea cruris* (*n* = 2, 1.3%), and *tinea faciei* (*n* = 1, 0.6%). The remaining 5 patients were affected by *tinea corporis* associated with onychomycosis (3.1%). Animal samples were collected by a veterinarian harvesting the hair surrounding the ringworm lesions using sterile clamps in symptomatic animals or brushing the asymptomatic ones following the MacKenzie modified technique [42].

Human mycological examination included direct light microscopy after 20% KOH clearing for the presence of fungal filaments and conidia and culture tests on Sabouraud peptone–glucose agar with and without cycloheximide, according to the standard techniques. Human mycological cultures were performed at the Dermatology Unit of Santa Maria Hospital in Terni, Dermatology Unit of Piero Palagi Hospital in Florence, Clinic Microbiology and Virology Laboratory of Papa Giovanni XXIII Hospital in Bergamo, and National Institute for Health, Migration and Poverty in Rome. Animal samples were analyzed at the Istituto Zooprofilattico Sperimentale of Umbria and Marche regions “Togo Rosati” (IZSUM). In practice, the samples were inoculated onto Dermasel agar, incubated at 25 ± 1 °C, and observed daily for up to 15 days. Fungal colonies attributable to *Trichophyton* spp. were evaluated basing on their macroscopic and microscopic features. Molecular techniques were carried out at the IZSUM to reach a reliable species identification. Therefore, colonies grown on Sabouraud or Dermasel agar were subjected to DNA extraction using the QIAamp DNA mini kit (QIAGEN^®^, Hilden, Germany) following a modified Gram-positive protocol (Appendix D: Protocols for Bacteria, Isolation of genomic DNA from gram-positive bacteria). The PCR amplification was performed using universal fungal primers ITS1 (5′-TCCGTAGGTGAACCTGCGG-3′) and ITS4 (5′-TCCTCCGCTTATTGATATGC-3′) [27]. In detail, PCR amplification was carried out on a total volume of 50 µL. The reaction mixture was prepared as follows: 5XGreen GoTaq^®^ Flexi Buffer (Promega, Madison, WI, USA), 2.5 mM of MgCl_2_ (Promega, Madison, WI, USA), 0.2 mM of each dNTP (Global Life Sciences Solutions Operations, Little Chalfont, UK), 0.4 mM of each primer, 1.25 units of GoTaq^®^ Hot Start Polymerase (Promega, Madison, WI, USA), 3 µL of template DNA and Nuclease-Free Water (Thermo Fisher Scientific, Austin, TX, USA). PCR amplification was performed in a Mastercycler Nexus X2 (Eppendorf AG, Hamburg, Germany) set to the following conditions: denaturation at 95 °C for 5 min; 35 cycles of 94 °C for 45 s, 56 °C for 45 s, and 72 °C for 1 min; and a final extension at 72 °C for 10 min. The PCR products were analyzed by gel electrophoresis on a 2% agarose gel and observed using a Midori Green Advance DNA stain (NIPPON Genetics Europe GmbH^®^). PCR-positive products (around 700 bp) were purified using a QIAquick PCR Purification Kit (QIAGEN^®^). The quality and quantity of the PCR product were assessed photometrically using a Biophotometer (Eppendorf AG, Hamburg, Germany). Sequencing was performed using a BrilliantDye^TM^ Terminator v3.1 Cycle Sequencing Kit (NimaGen^®^, Nijmegen, The Netherlands), and the reactions were separated through a 3500 Genetic Analyzer (Applied Biosystem, Foster City, CA, USA). Consensus sequences were created by BioEdit Sequence Alignment Editor software v 7.2.5 and aligned in the Westerdijk Fungal Biodiversity Institute database. Recently, all the 172 *Trichophyton* strains were selected and additionally analyzed through DermaGenius^®^ Resistance Multiplex real-time PCR assay (Pathonostics^®^, Maastricht, The Netherlands) to detect the presence of the most common (Phe397Leu and Leu393Phe) along with the less prevalent (Leu393Ser, Phe397Ile, and Phe397Val) *SQLE* gene mutants that confer terbinafine resistance [43]. Terbinafine resistance or susceptibility was evaluated analyzing the T_m_: T_m_ ranging between 54.0 and 64.0 °C is associated with terbinafine-resistant strains (mutant *SQLE* gene), while T_m_ ranging between 64.5 and 68.0 °C is associated with terbinafine-susceptibility strains (wild-type *SQLE* gene). Moreover, DermaGenius^®^ Resistance Multiplex real-time PCR assay allowed comparing the results obtained by Sanger sequencing.

## 3. Results

### 3.1. Human and Animal General Information

Human strains were obtained from 161 patients visited in the four microbiological centers participating in this retrospective study. The emerging species *T. indotineae* was isolated and identified from 12 patients (8 males and 4 females, median age 33 years) **who** came from Bangladesh (*n* = 7), Peru (*n* = 2), India (*n* = 1), Italy (*n* = 1), and Sri Lanka (*n* = 1) (Table 1).

Out of the 161 total strains, nine human samples were terbinafine-resistant. In detail, 1/161 (0.62%) was identified as *T. mentagrophytes* collected in 2016, 2/161 (1.24%) as *T. rubrum* and *T. indotineae* collected in 2023, and the remaining 6/161 (3.73%) as *T. indotineae* identified on May 2024.

All the 11 animal strains were identified as *T. mentagrophytes* from six Eurasian red squirrels, three dogs, one Eastern gray squirrel, and one cat.

Further details regarding macro and microscopical features and molecular issues will be examined in the following paragraphs.

### 3.2. Mycological Examination

The identification of *Trichophyton* strains was based on macroscopic and microscopic aspects of the colonies [44]. Culture characteristics such as surface texture, topography and pigmentation are variable and are therefore the least reliable criteria for identification. Microscopically, microconidia and macroconidia evaluation was based on the number, shape, and wall aspect as well as hyphae features [45].

### 3.3. Trichophyton Species Identification, Results by Sanger Sequencing

Sequence alignment showed reliable results considering overlap, similarity, and probability values. In detail, Sanger sequencing identified the 172 *Trichophyton* strains as follows: 88 (51.2%) *T. interdigitale*/*T. mentagrophytes* complex, 60 (34.9%) *T. rubrum*, 12 (6.9%) *T. indotineae*, 9 (5.2%) *T. tonsurans*, 2 (1.2%) *T. violaceum*, and 1 (0.6%) *T. verrucosum*, confirming at least the genus hypothesized through conventional mycological techniques. All the 11 animal strains belonged to the *T. interdigitale*/*T. mentagrophytes* complex. Considering that they differ for only a few SNPs in the ITS region [19], the reliable identification of the *T. interdigitale*/*T. mentagrophytes* complex and *T. indotineae* was achieved comparing the obtained consensus sequences with two *T. interdigitale* strains (LC508732 and LC508731) and the *T. indotineae* LC508728 strain [18]. *Trichophyton indotineae* sequences were deposited in the GenBank database under the accession numbers listed in Table 1.

### 3.4. Trichophyton Species Identification, Results by DermaGenius^®^ Resistance Multiplex Real-Time PCR Assay

All the 11 animal strains included in this study tested terbinafine sensitive. Among human strains, 152/161 (94.4%) showed terbinafine sensitivity and 9/161(5.6%) terbinafine resistance: one strain isolated in 2016 belonged to the *T. interdigitale*/*T. mentagrophytes* complex, two strains isolated in 2023 were found to be *T. indotineae* and *T. rubrum*, respectively, and six strains isolated in 2024 tested *T. indotineae*. Moreover, a DermaGenius^®^ Resistance Multiplex real-time PCR assay confirmed the results obtained by Sanger sequencing for all strains except for *T. verrucosum*.

**Figure 1:**
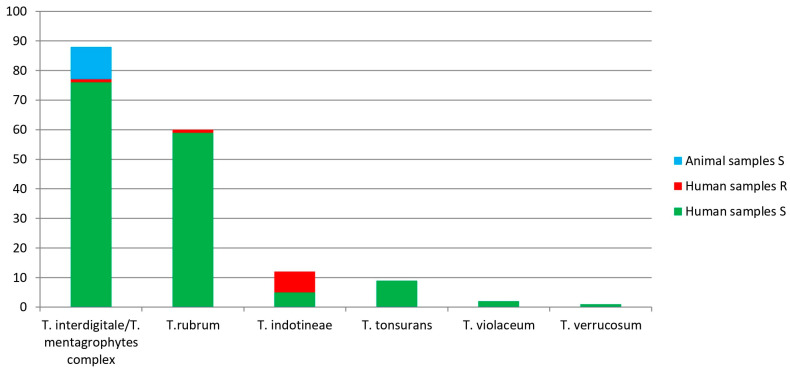
Dermatophyte species obtained by Sanger sequencing and their genotypic terbinafine sensitivity/resistance (S/R) analyzed by DermaGenius^®^ Resistance Multiplex real-time PCR assay in human samples (HS) and animal samples (AS).

The results obtained are shown in Figure 1 and detailed in Table S1.

## 4. Discussion

The epidemiological survey conducted in this study was able to identify retrospectively 172 *Trichophyton* strains isolated from humans (*n* = 161) and animals (*n* = 11) in Italy between January 2016 and May 2024. DermaGenius^®^ Resistance Multiplex real-time PCR assay confirmed the results obtained by Sanger sequencing for all strains except for one *T. verrucosum* strain, because it is not included in the DermaGenius panel. Moreover, DermaGenius^®^ Resistance Multiplex real-time PCR assay detected terbinafine resistance in 9 human strains (T_m_ 54.0–64.0 °C) and terbinafine susceptibility in the remaining 152 human and 11 animal strains (T_m_ 64.5–68.0 °C). Species identification was obtained combining conventional and molecular techniques particularly for the strains belonging to *T. indotineae*. *Trichophyton indotineae* indeed shows macro- and microscopical features similar to the *T. interdigitale*/*T. mentagrophytes* complex differing for only nine SNPs in the ITS region. A correct identification is crucial from an epidemiological point of view considering the rise in migratory flows from countries where this type of infection is endemic and antifungal resistance is common, mainly for terbinafine which is the drug of choice for the treatment of dermatophytoses. It is also important because the dermatophytes misidentification underestimates the real amount of cases.

Learning about terbinafine behavior in the *Trichophyton* strains allows for obtaining epidemiological information and avoiding unnecessary pharmacological treatment in drug-resistant strains. Unfortunately, in the clinical practice, this specific test is just performed in specialized laboratories, only in not responsive patients. As a result, epidemiological data could be underestimated, and precious time could be wasted regarding the improvement of the patients’ clinical conditions.

Moreover, by the Ministerial Decree 7 March 2022, the dermatophytosis cases must be notified (ICD9-CM code 110) regardless of the antifungal susceptibility or resistance. Case notification is another essential tool in order to distribute reliable data through Ministerial reports and to apply evidence-based public health interventions. The authors notified nine human terbinafine-resistant cases of *T. mentagrophytes* collected in 2016, two *T. rubrum* and 1 *T. indotineae* collected in 2023, and six *T. indotineae* identified on May 2024.

The results obtained in this study could highlight an increasing trend for *T. indotineae*, considering the seven terbinafine-resistant strains together with the five sensitive strains isolated in the current year. However, these strains were collected from patients from endemic areas for *T. indotineae* or having direct contacts with populations living in these areas and checked up in the National Institute for the promotion of migrant populations health. In fact, patients affected by *T. indotineae* came from Bangladesh (*n* = 7), Peru (*n* = 2), India (*n* = 1), Sri Lanka (*n* = 1), and Italy (*n* = 1). Considering the patients’ medical history, the authors hypothesized that the infections could have arisen in their native country. As for the Italian case, the medical history reported intimate contacts with an Indian person affected by *T. indotineae*. The results obtained from our study reinforce the emerging pathogenic role of *T. indotineae* in dermatophytoses, which was found predominantly in the Indian subcontinent and rapidly spread to other geographic areas.

As concerns animal samples, all 11 strains belonged to the *T. interdigitale*/*T. mentagrophytes* complex and tested terbinafine sensitive. Despite their limited number, data obtained about the terbinafine susceptibility were a hopeful sign considering the zoonotic role of *T. mentagrophytes*, particularly in pets.

The Italian medical community might be unprepared for the increase in dermatophyte cases, which is mainly due to the rise in migratory flows from countries where this type of infection is endemic, and diverse strains and antifungal resistance is common. In 2021, 2.3 million immigrants entered the European Union (EU) with an increase of almost 18% compared to the previous year [46]. On January 2022, over 5% of the almost 450 million people living in the EU were non-EU citizens. Systematic reviews revealed major knowledge gaps on the global burden of fungal infections and antifungal resistance especially related to morbidity data. Furthermore, fungal pathogens distribution and epidemiology can vary significantly by region. Our study raises awareness on the importance of microbiological identification of dermatophytes as well as case notification in order to collect epidemiological data and inform consequently dermatologists, general practitioners (GP), pediatricians, and veterinaries. Diagnostic challenges should be addressed together with specific professional training. An incorrect diagnosis and/or ineffective therapy can cause not only a delay in the patient’s recovery but also contribute to the spread of these pathogens and increase the cost of healthcare with broader public health implications. In recent times, Italy has already experienced severe outbreaks due to a hospital-acquired fungal infection [47]. A coordinated public health response must be implemented to prevent the widespread transmission of dermatophytes in community settings, i.e., refugee camps, schools, and gyms, which could even aggravate the burden of healthcare-associated infections, which is already serious in Italy. We recommend case notification to monitor trends in infections and antifungal resistance, the implementation of national clinical guidelines to manage dermatophytosis effectively and prevent human and animal transmission, specialized training for healthcare providers, and the availability of proper diagnostic tools as well as identification of regional reference laboratories. Additionally, public health campaigns aimed at educating the public about the prevention and early recognition of dermatophyte infections can mitigate their impact on drug-resistant communicable diseases in Italy and beyond. Indeed, on October 2022, the World Health Organization (WHO) published the first fungal priority pathogens list (WHO FPPL) [48] to highlight fungi of critical importance to human health, driving further research and policy interventions. The WHO FPPL list divided fungal pathogens into critical, high and medium priority categories. The main actions recommended were to strengthen laboratory capacity and surveillance, provide sustainable investments in research and development (R&D) and apply public health interventions. However, as for R&D investments, globally, the amount of public and private funding dedicated to fungal pathogens decreased substantially in the last few years (ranging from around 0.1 billion USD in 2017 to around 0.06 billion in 2021), while funding dedicated to bacterial pathogens increased in the same period (ranging from around 1.4 billion USD in 2017 to around 1.6 billion in 2021) [49]. Concerningly, R&D investments dedicated to bacterial or fungal pathogens amounted to about 85% or 5% of the overall funding for 2017–2021, respectively.

## 5. Conclusions

To the authors’ knowledge, there are no similar studies highlighting the importance of dermatophytoses in Italy in terms of antifungal resistance and the One Health concept. Clinicians as well as veterinarians may need to be informed carefully through specific education with the aim to (i) reduce diagnostic mistakes, (ii) provide effective therapy, (iii) improve the quality of patients’ life, (iv) prevent dermatophyte transmission and drug-resistance spread, (v) avoid underestimation of the actual burden of disease, and (vi) provide national recommendations.

In Italy, dermatophytoses are still considered mild infections despite a significant impact in the quality of patients’ life causing different kinds of disturbances and repeated clinical examinations. Just improving the clinical approach could overcome the limitations above mentioned to preserve the public health. Therefore, a correct information and diagnosis combined to a prompt pharmacological treatment can limit transmission, including the eventual antimycotic resistance spread.

The increasing resistance trend of *Trichophyton* strains leads the authors to further investigate other human and animal cases following the same One Health approach.

## Figures and Tables

**Table 1:** Information on the *Trichophyton indotineae* affected patients and the sequences deposited in the GenBank database.

Case Number ^1^	Year	Sex	Age	Native Country	Clinical Manifestation	Terbinafine S/R ^2^	Accession Number	Reference
105	2023	F	42	India	*Tinea corporis* and onychomycosis	S	OR192943	Crotti et al. 2023 [41]
115	2023	F	33	Sri Lanka	*Tinea corporis* and *tinea unguium*	R	OR880561	This study
147	2024	F	44	Peru	*Tinea pedis*	S	PP898430	This study
148	2024	M	16	Peru	*Tinea pedis*	S	PP898431	This study
149	2024	M	18	Bangladesh	*Tinea corporis*	R	PP898432	This study
150	2024	M	38	Bangladesh	*Tinea corporis*	R	PP898433	This study
152	2024	M	47	Bangladesh	*Tinea cruris*	S	PP898434	This study
153	2024	F	39	Bangladesh	*Tinea corporis*	R	PP898435	This study
156	2024	M	20	Bangladesh	*Tinea faciei*	R	PP898436	This study
159	2024	M	38	Bangladesh	*Tinea corporis*	S	PP898437	This study
170	2024	M	28	Bangladesh	*Tinea cruris*	R	PP898438	This study
171	2024	M	36	Italy	*Tinea cruris*	R	PP898439	This study

^1^ Further information is shown in Table S1. ^2^ S: terbinafine sensitive; R: terbinafine resistant.

## Data Availability

The data provided do not allow for identifying the patients enrolled in this study.

## Supplementary Materials

The following supporting information can be downloaded at https://www.mdpi.com/article/10.3390/jcm13185493/s1, Table S1: Details of the 172 *Trichophyton* analyzed strains: results of Sanger sequencing and DermaGenius^®^ Resistance Multiplex real-time PCR assay.
