# Supplementary material for: Terbinafine Resistance in Trichophyton Strains Isolated from Humans and Animals: A Retrospective Cohort Study in Italy, 2016 to May 2024
Source: J Clin Med. 2024 Sep 17;13(18):5493. doi: 10.3390/jcm13185493 (PMC11432958; doi:10.3390/jcm13185493)
Supplement: Supplementary file 1 [file jcm-13-05493-s001.zip › jcm-3193393-supplementary.pdf]

Table S1. Details of the 172 *Trichophyton* analyzed strains: results of Sanger sequencing and DermaGenius® Resistance Multiplex real-time PCR assay.

Case number[1]	Year	Sex	Age	Native Country	Clinical manifestation	Terbinafine S/R[2]	Accession number	Reference
105	2023	F	42	India	*Tinea corporis* and onychomycosis	S	OR192943	Crotti et al. 2023 [41]
115	2023	F	33	Sri Lanka	*Tinea corporis* and *tinea unguium*	R	OR880561	This study
147	2024	F	44	Peru	*Tinea pedis*	S	PP898430	This study
148	2024	M	16	Peru	*Tinea pedis*	S	PP898431	This study
149	2024	M	18	Bangladesh	*Tinea corporis*	R	PP898432	This study
150	2024	M	38	Bangladesh	*Tinea corporis*	R	PP898433	This study
152	2024	M	47	Bangladesh	*Tinea cruris*	S	PP898434	This study
153	2024	F	39	Bangladesh	*Tinea corporis*	R	PP898435	This study
156	2024	M	20	Bangladesh	*Tinea faciei*	R	PP898436	This study
159	2024	M	38	Bangladesh	*Tinea corporis*	S	PP898437	This study
170	2024	M	28	Bangladesh	*Tinea cruris*	R	PP898438	This study
171	2024	M	36	Italy	*Tinea cruris*	R	PP898439	This study
